# Measuring Asymmetric Ionic Current Waveform Through Micropores for Detecting Reduced Red Blood Cell Deformability Due to *Plasmodium falciparum* Infection

**DOI:** 10.3390/s25154722

**Published:** 2025-07-31

**Authors:** Kazumichi Yokota, Ken Hirano, Kazuaki Kajimoto, Muneaki Hashimoto

**Affiliations:** Health and Medical Research Institute, National Institute of Advanced Industrial Science and Technology (AIST), 2217-14, Hayashi-cho, Takamatsu 761-0301, Kagawa, Japan; kazumichi-yokota@aist.go.jp (K.Y.); hirano-ken@aist.go.jp (K.H.); k-kajimoto@aist.go.jp (K.K.)

**Keywords:** microfluidic sensing devices, RBC deformability, malaria, multi-physics simulation

## Abstract

**Highlights:**

**What are the main findings?**

- A microfluidic RBC deformability sensor was developed using the resistive pulse method.

**What is the implication of the main finding?**

- The asymmetric waveform detected by the sensor may be a reliable indicator of reduced deformability.
- This novel sensor allows for a detailed single-cell analysis of malaria-associated deformability reduction.

**Abstract:**

The mechanisms underlying reduced deformability of red blood cells (RBCs) in *Plasmodium falciparum* remain unclear. The decrease in RBC deformability associated with malarial infection was measured using ektacytometry, and only mean values were evaluated. In this study, we report the development of a microfluidic sensing device that can evaluate decreased RBC deformability at the single-cell level by measuring ionic current waveforms through micropores. Using an in vitro culture system, we found that when RBC deformability was reduced by *P. falciparum* infection, ionic current waveforms changed. As RBC deformability decreased, waveforms became asymmetric. Computer simulations suggested that these waveform parameters are largely independent of RBC size and may represent a reliable indicator of diminished deformability. This novel microfluidic RBC deformability sensor allows for detailed single-cell analysis of malaria-associated deformability reduction, potentially aiding in elucidating its pathology.

## 1. Introduction

Red blood cell (RBC) deformability, their ability to change shape, minimizes flow resistance and optimizes oxygen delivery to tissues. Reduced deformability results in impaired perfusion and oxygen delivery to peripheral tissues, and rigid RBCs can directly block capillaries [1,2]. Reduced RBC deformability has been implicated in circulatory disorders and anemia observed in diverse pathologies, such as thalassemia, sickle anemia, sepsis, diabetes, cardiovascular conditions, and stroke [3,4,5,6,7].

*Plasmodium falciparum,* the causative agent of the most severe forms of malaria, infects RBCs and undergoes various developmental stages, including ring formation, trophozoite development, and schizont production. After nuclear division, merozoites that invade new RBCs are generated upon release into the blood. In *P. falciparum* malaria, the deformability of infected and uninfected RBCs is reduced, contributing to reduced RBC flow through the microvasculature and impaired organ perfusion [8,9].

The association between malaria infection and reduced RBC deformability has been evaluated using ektacytometry [10,11,12,13,14]. This can be attributed to the high detection sensitivity of ektacytometry [15]. In contrast, ektacytometry can detect only an average reduction in RBC deformability. By measuring the reduction in deformability at the single RBC level, we can clarify the details of the reduction in RBC deformability caused by malaria and contribute to elucidating the pathology of the disease. Several devices that measure deformability at the single RBC level are available, including micropipette aspiration, optical tweezers, and atomic force microscopy (AFM). These assays require skilled operators and have low throughput, typically measuring only a few dozen RBCs per test [16].

The resistive pulse method (RPM) probes individual nano- to micro-sized particles as pulse-like electrical signals. The translocation of a particle passing through a pore can be evaluated as a transient ionic current blockade. The measured ionic current blockade signals contain information regarding the physical properties of the particles, such as their size, shape, surface charge, and elastic deformability [17,18,19,20,21,22,23].

In this study, we present a microfluidic RBC deformability sensor that analyzes the reduced RBC deformability during *P. falciparum* infection. By utilizing the RPM principle, the sensor evaluates single-cell structural deformation as RBCs pass through pores. The sensor may provide deeper insights into the link between malaria severity and reduced RBC deformability, ultimately advancing our understanding of malaria pathology.

## 2. Materials and Methods

### 2.1. Multiphysics Simulations of Ionic Current Waveform for RBCs with Different Membrane Stiffness

The structural deformation of RBC during translocation through a micropore was numerically simulated by solving the fluid–structure interaction using the finite-element method. The fluid flow and structural mechanics are described by the (1) Navier–Stokes equation and (2) momentum equation as follows:

(1) $$\rho_{f}\left(\frac{\partial u}{\partial t}+\left(u\cdot \nabla \right)u\right)=-\nabla \left[pI+\mu \left(\nabla u+{\left(\nabla u\right)}^{T}\right)\right],\;\rho_{f}\nabla \cdot u=0$$

and

(2) $$\rho_{s}\frac{\partial v}{\partial t}=\nabla \cdot \sigma +f$$

Here, *ρ*_f_, *u*, *p*, and *μ* are density, velocity field, pressure, and viscosity, respectively, for the fluid; *ρ*_s_, *v*, *σ*, and *f* are density, velocity filed, stress tensor, and force at unit area, respectively, for the solid membrane, where I denotes the unit diagonal matrix. The interaction between the fluid and solid membrane was denoted by the balance of the normal force per unit area on the surface using the following equation:

(3) Γ·n=σ·n

where $\Gamma =-pI+\mu (\nabla u+{\left(\nabla u\right)}^{T})$, and *n* is the normal vector.

**Figure 1:**
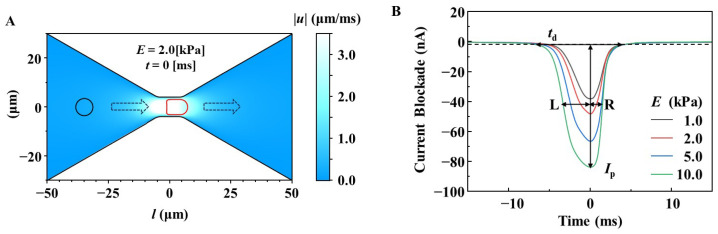
(**A**) Simulation model of an RBC passing through a micropore with a diameter of 8.0 μm and a length of 10.0 μm. The RBC is assumed as a spherical membrane with a diameter of 7.5 μm. (**B**) Simulated waveforms for the translocation of the RBC models with different Young’s moduli. Note that as the Young’s modulus increases, pulse height (*I*_p_), duration time (*t*_d_), and asymmetry (L/R ratio) increase.

The micropore was modeled as the constriction, of which diameter and length were 8.0 and 10.0 μm, in the microchannel (Figure 1A). The diameter and length of the simulated microchannel was 60 and 100 μm, and the fluid inflow and outflow were set on both ends of the microchannel (left and right sides of Figure 1A). In the simulation of fluid filled in the pore and microchannel, we used a density of *ρ*_f_ = 1.00 g/cm^3^ and a viscosity of *μ =* 1.01 mPa·s, which correspond to those of water at room temperature, for solving Equation (1). The boundary conditions of the pressure in the inflow and outflow of the microchannel were *p*_in_ = 25 Pa and *p*_out_ = 0 Pa, respectively. A spherical membrane 7-μm in diameter was placed 15 μm from the inflow end, depicted by the black circle in Figure 1A, as the initial position of the RBC model. Assuming that the RBC membrane was a linear elastic membrane, we systematically investigated various Young’s moduli for the membrane as models with different stiffness values for the RBC. Here, Young’s modulus (*E*) is associated with the stress (*σ*) and strain (*ε*) and expressed as *σ* = *Eε.* Furthermore, we used *ρ*_f_ of 1.1 g/cm^3^, a thickness of 30 nm, and Poisson’s ratio of 0.49 for the membrane and simultaneously solved the equations under the boundary conditions of free deformation for the membrane in Equation (2), and no fluid flows across the membrane, as shown in Equation (1).

The time-dependent fluid–solid interaction was solved using Equations (1)–(3), and the translocation of the RBC model from the inflow side to the outflow side through the micropore (illustrated by the broken line arrows in Figure 1A) with a pressure difference of *p*_in_–*p*_out_ = 25 Pa was simulated. During translocation, structural deformation of the RBC model (depicted by the red circle in Figure 1A) was observed. The electrical resistances (*R*) of the microchannel and micropore were calculated from the simulated results of the structural deformation using the following equation:

(4) $$R(t)=\frac{1}{\kappa }\int \frac{dl}{A(t,l)}$$

Here, *κ* and *A* were the electrical conductivity and the cross-sectional area of the fluid outside the spherical membrane at *t*. We used *κ* = 1.73 S/m, assuming phosphate-buffered saline (PBS). The electrical current waveform *I* (*t*) (Figure 1B) was obtained using *I* (*t*) = *V*_b_ /(*R* (*t*) + 2*R*_acc_), where *V*_b_ is the applied bias voltage for the ionic current measurement. *R*_acc_ is the electrical resistance outside the simulation model in the microfluidic RBC deformability sensor, which is fabricated and used for measurements. It describes the resistance between electrodes and a pore, referred to as access resistance. We used *R*_acc_ = 4 MΩ. All the simulations were performed using COMSOL Multiphysics version 6.3 (Stockholm, Sweden).

### 2.2. Parasite Culture

*P. falciparum* (3D7 strain) was cultured as previously described [24]. We synchronized the parasites at the ring or schizont stage using 5% d-sorbitol and highly synchronized the parasite-infected RBCs (>95%) [25]. Parasitemia and parasite stages were determined by microscopic analysis of Giemsa-stained thin blood films. Parasitemia was estimated by counting over 3000 RBCs (% parasitemia = [parasitized RBCs/total RBCs] × 100).

### 2.3. Preparation of Microfluidic RBC Deformability Sensor

**Figure 2:**
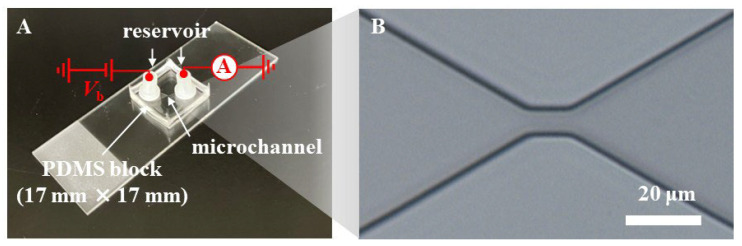
(**A**) Photographic image of a microfluidic RBC deformability sensor. A 17 mm × 17 mm PDMS block with a microchannel was bonded to a slide glass, and the microchannel was connected to reservoirs via through-holes. The ionic current was measured via the electrodes inserted into the inlet and outlet reservoirs at a constant bias voltage of *V*_b_ = 3.0 V. (**B**) Microscopic image of a micropore formed by a constriction within the microchannel. Scale bar = 20 μm.

Microfluidic RBC deformability sensors (Figure 2A) were prepared as previously described, with certain modifications [26]. A microchannel was formed on a 17 mm × 17 mm polydimethylsiloxane (PDMS) block and connected to inlet and outlet reservoirs through holes. In this study, the height of the microchannels was 7 µm, a constriction (8 μm in width and 10 μm in length at the narrowest region) as a micropore was fabricated within the microchannel (Figure 2B), and nylon reservoirs (Nylon Screw Insulator, M3, 6 mm, RS Group plc, London, UK) with an inner diameter of 3.1 mm were used. The PDMS block was then bonded to a slide glass.

### 2.4. Ionic Current Measurement

Prior to measurements, the microchannels in the fabricated microfluidic RBC deformability sensor were filled with PBS as an electrolyte solution. Subsequently, 10 μL of RBC samples suspended in PBS (2–5 × 10^6^ RBCs/mL) were injected through the inlet. For each measurement, 19 μL of PBS was added to the inlet reservoir to apply a pressure of 25 Pa as a driving force for RBCs in the microchamber from the inlet to the outlet. The flow rate through the micropore at this pressure was estimated to be ~0.2 nL/s from simulations. A pair of Ag/AgCl ink (ALS Co., Ltd., Tokyo, Japan) -coated platinum (φ = 0.8 mm and 99.98% purity, The Nilaco Corporation, Tokyo, Japan) was used as electrodes and inserted into the inlet and outlet reservoirs. A constant bias voltage of *V*_b_ = 3.0 V (transient response time was set to normal, and root mean square (RMS) noise was 1.4 mV) was applied to the electrode at the outlet, while the inlet electrode was grounded (Figure 2A). The ionic current was measured using a source measurement unit (SMU, NI PXIe-4141, National Instruments, Austin, TX, USA) over a current range of 10 µA (RMS noise was 4.2 nA). The SMU was controlled using LabVIEW software (LabVIEW 2020, National Instruments). Time traces of the ionic current were recorded at a sampling rate of 50 kHz, which corresponds to a resolution time of 20 µs. During electrical measurements, the translocation of the RBC through the micropores was observed using an inverse optical microscope (DIML II; Leica Camera AG, Wetzlar, Germany).

### 2.5. Statistical Analysis

Statistical analyses were performed using Sigma Plot version 16.0 Software (Systat Software, Inc., San Jose, CA, USA) with one-way ANOVA (Tukey’s test). Unless otherwise stated, sample sizes of 50 RBCs were used for statistical analysis.

## 3. Results and Discussion

### 3.1. Design of Microfluidic RBC Deformability Sensors for Measuring RBC Deformability Using a Multi-Physics Simulation Approach

The width and length of the micropores in microfluidic RBC deformability sensors are important for evaluating the physical properties of the cells of interest. As the diameter of RBCs is approximately 7–8 μm, smaller than that of cancer cells and white blood cells, we determined that the micropore size for RBC deformability sensing should be smaller than those used in our previous studies on cancer cells and white blood cells [26]. However, significantly narrow widths or extended lengths often lead to clogging of RBC micropores; therefore, a wider and shorter pore design is preferable for high-throughput single-cell measurements.

Simulations were performed to design the microfluidic RBC deformability sensor (Figure 1A). We designed micropore structures that can detect current blockade waveform patterns corresponding to a decrease in RBC deformability using RBC models with various levels of deformability. The stiffness of the simulated RBC was set to Young’s modulus of *E* = 1.0, 2.0, 5.0, and 10.0 kPa, and the waveform patterns for various micropore structures were compared. When the micropore diameter was 8.0 µm and the length was 10.0 μm, the following waveform characteristics were observed.

When the RBC deformability was high at *E* = 1 kPa, the pulse shape had a small height of current blockade (*I*_p_) and a short duration to pass through the micropores (*t*_d_), and the left and right widths from the peak top of the waveform (L and R) were almost the same, with the ratio (L/R ratio) being close to 1 (Figure 1B). As RBC deformability decreased with an increase in *E*, *I*_p_ and *t*_d_ increased, which is consistent with previously reported results [22,27]. In this study, we found that the waveform became asymmetric with an L/R ratio greater than 1, and that this value increased proportionally with decreasing RBC deformability.

### 3.2. Simulation of the Effect of RBC Size on the Changes in I_p_, t_d_, and L/R Ratio

The evaluation of physical properties using the RPM is strongly influenced by the size of the object being measured. The size of RBCs is not uniform, and *P. falciparum* infection is reported to affect RBC size [28].

**Figure 3:**
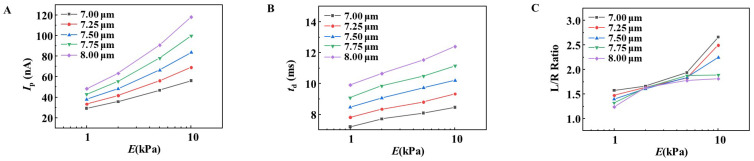
Simulation of the effect of RBC size on *I*_p_, *t*_d_, and L/R ratio. The RBC deformability sensor simulates *I*_p_ (**A**), *t*_d_ (**B**), and L/R ratio (**C**) for materials with various deformability of 7, 7.25, 7.5, 7.75, and 8 μm. Note that the L/R ratio increases in a deformability-dependent manner, regardless of the size of the material being measured.

Considering that the size of RBCs is 7–8 μm in diameter, we simulated and compared the *I*_p_, *t*_d_, and L/R ratios of objects with various deformability with diameters of 7.00, 7.25, 7.50, 7.75, and 8.00 μm (Figure 3). As shown in Figure 3A,B, the values of *I*_p_ and *t*_d_ increased with the increase in the diameter of the RBC model, even if the deformability was the same. Therefore, when the size of the RBC being measured is not uniform, an increase/decrease in *I*_p_ or *t*_d_ indicates a decrease/increase in deformability, as well as reflects the size.

In contrast, the L/R ratio was less affected by the size of the RBCs on deformability and increased depending on the decrease in deformability when the *E* value was 5 kPa or less (Figure 3C). Ciasca et al. reported that the average *E* value for RBC was 1.82 ± 0.2 kPa [29]. In addition, because the L/R ratio of *P. falciparum*-infected RBCs rarely exceeds 1.7 (see Figure 3), an *E* value exceeding 5 kPa was considered to be extremely rare. These results suggest that RBC deformability using RPM should be evaluated using the L/R ratio.

### 3.3. Measurement of Deformability of P. falciparum-Infected RBCs Using a Microfluidic RBC Deformability Sensor

Using this microfluidic RBC deformability sensor, we measured parasite-infected RBCs that were synchronously cultured in vitro. Healthy RBCs (H-RBCs) that were not infected with parasites were used as negative controls. The sensors measured RBCs infected with the ring-form parasites (R-RBCs), expected to minimally affect deformability owing to their small volume, and schizonts-infected RBCs (S-RBCs), anticipated to significantly reduce deformability because of the larger parasite load [30].

**Figure 4:**
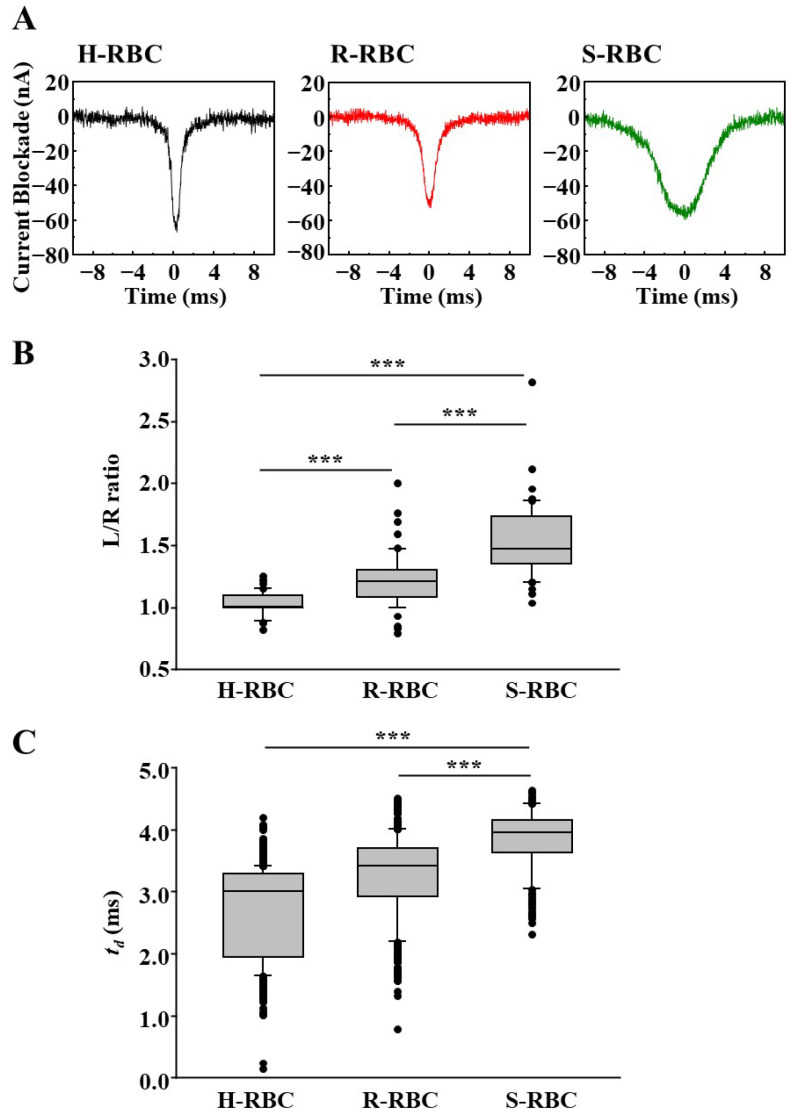
RBC deformability of H-RBCs, R-RBCs, and S-RBCs. (**A**) Typical waveform of the ionic current blockade by RPM was observed for healthy uninfected RBC (H-RBC), ring form-infected RBC (R-RBC), and schizont-infected RBC (S-RBC). L/R ratio (**B**) or *t_d_* (**C**) was calculated for 50 randomly selected RBCs in each group; boxes, interquartile range; horizontal lines, median. *** *p* < 0.001.

Figure 4A shows the typical waveforms detected by the microfluidic RBC deformability sensors. The H-RBCs have sharp waveforms and appear symmetrical. R-RBCs had waveforms with L/R ratios greater than 1. The S-RBCs exhibited waveforms with a significant increase in the L/R ratio. To examine the statistical significance of the results, multiple waveforms were compared and analyzed (Figure 4B). We randomly selected 50 waveforms from each RBC group and manually measured their L/R ratios. The L/R ratio was significantly higher in R-RBCs compared to H-RBCs, and even greater in S-RBCs than in R-RBCs.

Previously, decreased RBC deformability due to parasite infection was evaluated based on the increase in *t_d_* using RPM [31]. In this study, we investigated whether *t_d_* was longer in R- and S-RBCs than in H-RBCs (Figure 4C). The *t_d_* of S-RBCs was significantly longer than that of S- or R-RBCs. In contrast, the *t_d_* of the R-RBCs was not significantly longer than that of the H-RBCs (*p* = 0.31). This indicates that the decrease in RBC deformability due to ring-form infection requires a higher detection sensitivity of the sensor than the decrease in RBC deformability due to Schizont infection.

In previous studies, a reduction in RBC deformability owing to malaria was demonstrated and analyzed using ektacytometry. The objectives of this study were to develop a microfluidic RBC deformability sensor capable of high-throughput single-cell detection of reduced deformability, enabling detailed analysis and contributing to a deeper understanding of pathology. Our microfluidic RBC deformability sensor allowed for the measurement of deformability up to 30 RBC/min. Compared with measurement devices that measure at the single RBC level, such as AFM, this microfluidic RBC deformability sensor enables high-throughput measurements.

### 3.4. Detection of RBC with P. falciparum Inside Using Microfluidic RBC Deformability Sensors

**Figure 5:**
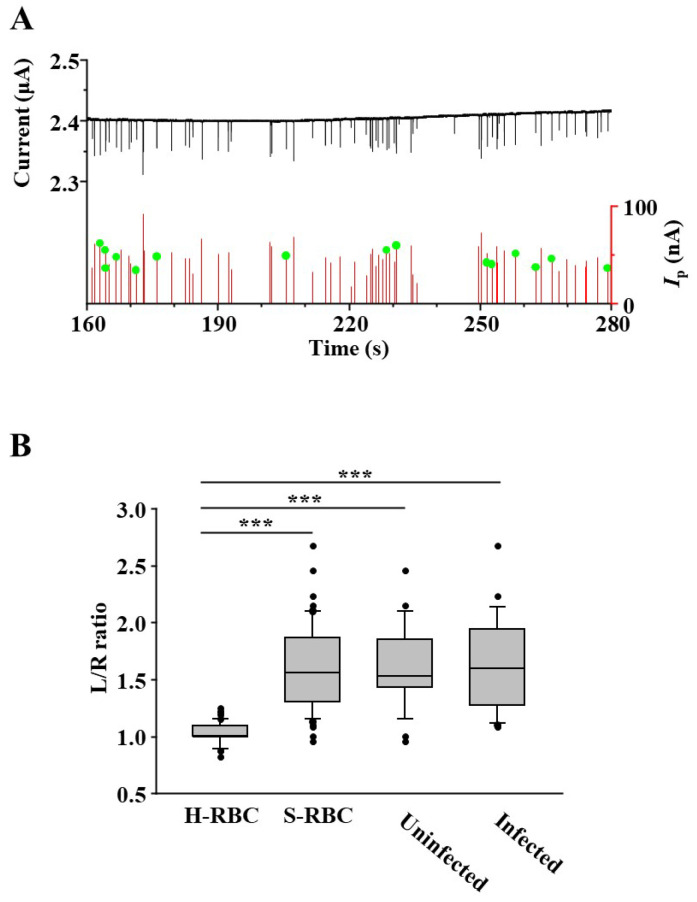
(**A**) Scattering plot of transit time versus ionic current dip for S-RBC. The waveform signals of detected RBCs are marked with red lines. The signals of RBCs with schizonts inside detected by fluorescence microscopy are marked with green circles. (**B**) L/R ratio of the waveforms for uninfected RBCs (uninfected) and RBCs with schizonts (infected) in the S-RBC group were compared and analyzed. *** *p* < 0.001.

We investigated the ability of this microfluidic RBC deformability sensor to detect parasitized RBCs from uninfected cells within a population of infected RBCs (Figure 5). Figure 5A shows the results of measuring S-RBCs using the microfluidic RBC deformability sensor. Before applying the S-RBCs to the microfluidic RBC deformability sensor, the S-RBCs were stained with acridine orange (AO), and the schizonts were fluorescently stained for nuclei. Schizonts exhibit polymorphism, resulting in strong fluorescent signals. A total of 142 RBCs were counted, and fluorescence microscopic images were videotaped. The waveform of RBCs infected with schizonts was identified by comparing the passage time of the fluorescent signal with the detected wavelength (Figure 5A, green circle).

Of all RBCs, 26 were infested with schizonts, resulting in an infection rate of 18.3%. This was close to the infection rate calculated using Giemsa staining and microscopic observations, indicating that S-RBCs could be accurately detected. Figure 5B shows the results of the comparison of the L/R ratio between RBCs with or without schizonts (infected or uninfected, respectively). No statistically significant differences were observed in either analysis, demonstrating that the microfluidic RBC deformability sensor could not detect RBCs with schizonts in the blood samples. The ring form had a weaker fluorescent signal in AO staining than the schizonts, and identifying them by videotaping under a fluorescent microscope was challenging. Thus, it may be difficult to apply this sensor clinically for malaria diagnosis by parasite detection.

Yang et al. developed a microfluidic RBC deformability sensor that could distinguish RBCs with the parasite inside from uninfected RBCs from a group of parasite-infected RBCs and calculate the infection rate [31]. By mimicking the micropore structure to resemble a capillary blood vessel, they succeeded in identifying RBCs inside the parasite within the time they took to pass through the pores (*t*_d_). Calculating the infection rate requires Giemsa staining. Therefore, the advantage of being able to calculate the label-free infection rate is useful. However, this microfluidic RBC deformability sensor could not detect a decrease in the deformability of uninfected RBCs in the parasite culture.

Infection with *P. falciparum* malaria, *P. vivax,* and *P. knowlesi* has been reported to reduce the overall deformability of RBCs, including non-infected RBCs, and this is closely related to the severity and pathology of the disease. Analysis of deformability at the single RBC level using microfluidic RBC deformability sensors may reveal important aspects of malaria pathology that are not revealed by ektacytometry. Barber et al. reported no significant differences in the mean RBC deformability between severe and non-severe cases of *P. falciparum* malaria [10]. However, capillaries become more susceptible to clogging as the condition worsens, and changes in RBC deformability may exhibit some differences. For example, patients with severe malaria may have a high proportion of extremely rigid RBCs, increasing the risk of microvascular obstruction, one of the key factors contributing to cerebral malaria.

## 4. Conclusions

We developed a novel microfluidic RBC deformability sensor that applies the principle of RPM. This sensor can specifically detect RBC deformability by measuring a new index (the L/R ratio) without being affected by RBC size. In the future, microfluidic RBC deformability sensors may enable single-cell RBC analysis from malaria patients with varying disease severities, potentially revealing new aspects of malaria pathology and contributing to efforts to overcome malaria.

## Data Availability

The original contributions presented in this study are included in the article. Further inquiries can be directed to the corresponding author.
